# Whole genome sequence data set of methicillin-resistant *Staphylococcus aureus* isolated from a milkman associated with cows with subclinical mastitis in Kiruhura district, Uganda

**DOI:** 10.1016/j.dib.2026.113196

**Published:** 2026-08-25

**Authors:** Reagan Muhwezi, Vidya Sankarapandian, GR. Neel, Emmanuel Eilu, Ambrose Shabohurira, Musoba Abubakar, Lutaya Festus David, Sulman Muhanguzi, Theophilus Pius, Naheem Adekilekun Tijani, Danladi Makeri

**Affiliations:** a Department of Microbiology and Immunology, Kampala International University Western Campus, P.O. Box 71, Bushenyi, Western Uganda, Uganda; b College of Veterinary Medicine, Animal Resources and Biosecurity (CoVAB), Makerere University, P.O. Box 7062, Kampala, Uganda; c Department of Medical Laboratory, Ndejje University, P.O. Box 7088, Kampala, Uganda

**Keywords:** Antimicrobial-resistant genes *Staphylococcus aureus*, Methicillin-resistant *staphylococcus aureus* (MRSA), Subclinical mastitis, Genomic annotation, MinION MK1B sequencing

## Abstract

The whole-genome sequence data set for methicillin-resistant *Staphylococcus aureus*, which was isolated from a milkman associated with cows with subclinical mastitis in the Kiruhura district of Uganda, is presented here. The assembled genome size was 2822,509 bp, with a 33% GC, 2 Contigs, a Contig N50 of 2818,424, and 1 Contig L50. You can access the genome sequence and related metadata at https://www.ncbi.nlm.nih.gov/datasets/genome/GCA_056782255.1/. This dataset can be used again for resistance gene mapping, comparing genomic analysis, and comprehending genetic diversity among MRSA isolates from Ugandan milkmen.

Specifications of the Table

**Table:** 

Subject	Health Sciences, Medical Sciences & Pharmacology
Particular field of study	Antimicrobial resistance, virulence characterization, and genomes of bacteria
Data type	Raw Sequence Reads (FASTQ), Assembled (FASTA), Annotated (GenBank format)
Data collection	A nasal swab was collected from a milkman and cultured on Mannitol Salt Agar. In accordance with CLSI recommendations, Kirby-Bauer disk diffusion was used for antimicrobial susceptibility testing. The NEB Monarch Spin gDNA Extraction Kit was used to extract the DNA, and the Qubit 3.0 Fluorometer and NanoDrop ND-1000 (Thermo Fisher Scientific) were used to evaluate the concentration and purity of the isolated DNA. The MinION MK1B platform was used for sequencing. Before and after trimming, the raw reads were quality checked using NanoPlot (v1.46.0), trimmed with NanoFilt, assembled by de novo assembly with Flye *(*v2.9.5+galaxy0), Medaka polisher (v 0.13.0) was used for polishing, QUAST (v5.3.0) was used to quality check the assembled reads, BUSCO (v5.8.3) determined the completeness of the assembly, which was submitted to NCBI and annotated with PGAP (v6.10), and CheckM analysis (v1.2.4) was also used to check for completeness.
Location of the data source	Kiruhura District, South-western, Uganda; −0.208,181, 30.843,584
Data availability	Publicly accessible data can be found in the NCBI repository: BioProject accession: PRJNA1450645 BioSample accession: SAMN57163795 Genome accession: JBXAVD000000000.1 Assembly accession: GCA_056782255.1 SRA accession: SRR38001508 Direct data URLs: https://www.ncbi.nlm.nih.gov/bioproject?term=PRJNA1450645 http://www.ncbi.nlm.nih.gov/biosample?term=SAMN57163795 https://www.ncbi.nlm.nih.gov/nuccore/JBXAVD000000000 https://www.ncbi.nlm.nih.gov/datasets/genome/GCA_056782255.1/ https://trace.ncbi.nlm.nih.gov/Traces/?run=SRR38001508
Related study article	None

## 1 The Data'S Value

• The genome provides insights into virulence determinants and antimicrobial resistance mechanisms underlying the pathogenic potential of MRSA among milkmen, which is critical for mastitis control and food safety.
• The dataset supports molecular epidemiology by enabling strain tracking, contributing to the limited region-specific dairy disease-related genomic resources.
• The sequence will facilitate comparative genomics of veterinary, clinical, and environmental *S. aureus* strains, particularly for analysing virulence profiles, resistance genes, and mobile genetic elements.
• Beyond microbiology, the dataset can be used in bioinformatics training and benchmarking of genome assembly, annotation, and algorithmic pipelines.

## 2 Background

Due to its asymptomatic nature, subclinical mastitis (SCM) presents serious problems for dairy farmers, resulting in food insecurity and financial losses in poor nations with limited access to sophisticated veterinary laboratory facilities [1]. *Staphylococcus aureus* is recognized as one of the most common microorganisms associated with contagious SCM, and when it is MRSA, the stakes for the community rise significantly [2]. A milkman remains an essential element in this cycle because MRSA is a persistent zoonotic pathogen, spread quickly through collectors' hands, containers, and equipment during the milking process, mostly as a result of poor hygiene [3]. A milkman may unintentionally become a vector in the absence of strict hygiene procedures, transferring resistant strains from one diseased herd to many healthy ones and ultimately to the customer [4]. This dataset provides genomic insights into resistance and virulence genes of MRSA from a milkman handling cows with subclinical mastitis in Uganda, Kiruhura district, and also offers valuable information for researchers studying antibiotic stewardship, disease evolution, and genetic surveillance.

## 3 Description of Data

The assembled genome statistics are as shown in Table 1. Check M (v1.2.5) analysis revealed completeness and contamination level, Average Nucleotide Identity (ANI) analysis revealed *Staphylococcus aureus* (GCA_006094915.1) as the closest type strain with an ANI, assembly coverage, type assembly coverage, confirming species-level identity and close relatedness as shared in Table 1. As indicated in Table 2, resistant genes were found using ResFinder and the Comprehensive Antibiotic Resistance Database (CARD). The associated virulence factors were found using the Virulence Factor Database (VFDB), which is presented in Table 3.

**Table 1:** Genome statistics, and features of methicillin-resistant *S. aureus.*

Assembly statistics	
Parameter	Value
Genome size	2822,509 bp
Total ungapped length	2822,509 bp
Number of contigs	2
Contig N50	2818,424 bp
Contig L50	1
GC percent	33
Genome coverage	2.87x
Largest contig	2818,424
Smallest contig	4085
**Parameter**	**Value**
Genes	2808
CDSs	2726
rRNAs	7, 6, 6 (5S, 16S, 23S)
tRNAs	59
ncRNAs	4
Quality analysis	
**Parameter**	**Value**
Completeness	92.93%
Contamination	0.4%
**BUSCO assembly metrics**	
Complete BUSCOs (C)	96.8%
Complete and single-copy BUSCOs (S)	96.85
Complete and duplicated BUSCOs (D)	0.0%
Fragmented BUSCOs (F)	3.2%
Missing BUSCOs (M)	0.0%
Total BUSCO groups searched (n)	124
**Average Nucleotide Identity (ANI) match details**	
**Parameter**	**Value**
Type assembly	GCA_057816525.1
Organism name	Staphylococcus aureus
ANI	98.16%
Assembly coverage	91.21%
Type assembly coverage	92.52%

**Table 2:** Resistant gene characteristics and features associated with the assembled genome.

Contig	Gene	%Coverage	%Identity	Database	Accession	Primary Class of Antibiotics / Resistance Target
contig_1	*ErmC*	100	99.86	CARD	M12730.1:779–1514	Lincosamides
contig_2	*mepA*	100	98.97	CARD	AY661734.1:839–2195	Fluoroquinolone and Glycylcyclines
contig_2	*mepR*	100	100	CARD	BA000017.4:379,931-380,351	Tetracycline and Glycylcyclines
contig_2	*tet(38)*	100	98.74	CARD	AY825285.1:0–1353	Tetracycline
contig_2	*norC*	99.86	98.49	CARD	CP005288.1:116,946-118,335	Fluoroquinolone (and Antiseptics)
contig_2	*mecA*	100	99.9	CARD	KC243783.1:0–2007	Beta-lactam (Penicillin)
contig_2	*sdrM*	99.93	81.04	CARD	BA000018.3:2241,223-2239,879	Fluoroquinolone (and Antiseptics)
contig_2	*sepA*	98.95	83.8	CARD	NC_016941.1:2187,613-2187,139	Fluoroquinolone (Antiseptics)
contig_2	*S_aureus_LmrS*	99.93	93.83	CARD	CP000046.1:2236,817-2235,374	Multi-class Efflux Pump (Aminoglycoside, Diaminopyrimidine, Macrolide, Oxazolidinone, Phenicol)
contig_2	*kdpD*	100	99.96	CARD	FHDU01000010.1:56,636-53,978	Aminoglycoside
contig_2	*PC1_beta-lactamase_(blaZ)*	100	96.45	CARD	CP000732.1:10,528–9682	Beta-lactam (Penicillin)
contig_2	*dfrC*	100	98.79	CARD	AE015929.1:1129,419-1128,933	Diaminopyrimidine (e.g., Trimethoprim)
contig_2	*AAC(6′)-Ie-APH(2′')-Ia*	100	100	CARD	GU565967.1:26,419-24,979	Aminoglycoside
contig_2	*arlR*	100	98.79	CARD	CP023390.1:1462,248-1461,588	Fluoroquinolone (and Antiseptics)
contig_2	*arlS*	100	98.82	CARD	CP000253.1:1361,636-1360,280	Fluoroquinolone (and Antiseptics)
contig_2	*S_aureus_norA*	99.91	92.2	CARD	D90119.1:477–1644	Fluoroquinolone
contig_1	*erm(C)_13*	100	100	Resfinder	M13761	Macrolide, Lincosamide, Streptogramin (MLS)
contig_2	*mecA_6*	100	100	Resfinder	BX571856	Broad Beta-lactam (Penicillins, Cephalosporins, Carbapenems)
contig_2	*blaZ_138*	100	100	Resfinder	CP003979	Beta-lactam (Penicillins)
contig_2	*aac(6′)-aph(2′')_1*	100	100	Resfinder	M13771	Aminoglycoside

**Table 3:** Virulence factor characteristics and features associated with the assembly.

Gene	Contig	% Coverage	% Identity	Database	Accession	Function
*Geh*	contig_2	100	97.93	VFDB	NP_645,114	enzymes and secretion
*essB*	contig_2	100	95.43	VFDB	NP_645,078	enzymes and secretion
*esaB*	contig_2	100	93.42	VFDB	NP_645,076	enzymes and secretion
*essA*	contig_2	100	99.78	VFDB	NP_645,075	enzymes and secretion
*esaA*	contig_2	99.97	98.78	VFDB	NP_645,074	enzymes and secretion
*Lip*	contig_2	100	98.58	VFDB	NP_647,407	enzymes and secretion
*Aur*	contig_2	100	90.33	VFDB	NP_647,375	enzymes and secretion
*sspA*	contig_2	96.04	96.93	VFDB	NP_645,749	enzymes and secretion
*sspB*	contig_2	100	99.24	VFDB	NP_645,748	enzymes and secretion
*sspC*	contig_2	100	99.7	VFDB	NP_645,747	enzymes and secretion
*hysA*	contig_2	98.73	92.85	VFDB	NP_646,946	enzymes and secretion
*esxA*	contig_2	100	99.32	VFDB	NP_645,073	enzymes and secretion
*cap8P*	contig_2	100	98.95	VFDB	NP_644,954	biofilm and capsule formation
*cap8O*	contig_2	100	98.73	VFDB	NP_644,953	biofilm and capsule formation
*cap8N*	contig_2	100	98.76	VFDB	NP_644,952	biofilm and capsule formation
*cap8M*	contig_2	100	99.64	VFDB	NP_644,951	biofilm and capsule formation
*cap8L*	contig_2	100	99.67	VFDB	NP_644,950	biofilm and capsule formation
*cap8G*	contig_2	100	99.38	VFDB	NP_644,945	biofilm and capsule formation
*cap8F*	contig_2	99.91	98.47	VFDB	NP_644,944	biofilm and capsule formation
*cap8E*	contig_2	100	99.03	VFDB	NP_644,943	biofilm and capsule formation
*cap8D*	contig_2	100	99.29	VFDB	NP_644,942	biofilm and capsule formation
*cap8C*	contig_2	100	99.35	VFDB	NP_644,941	biofilm and capsule formation
*cap8B*	contig_2	100	92.87	VFDB	NP_644,940	biofilm and capsule formation
*cap8A*	contig_2	100	97.29	VFDB	NP_644,939	biofilm and capsule formation
*icaC*	contig_2	100	97.25	VFDB	NP_647,406	biofilm and capsule formation
*icaB*	contig_2	100	99.43	VFDB	NP_647,405	biofilm and capsule formation
*icaD*	contig_2	100	99.67	VFDB	NP_647,404	biofilm and capsule formation
*icaA*	contig_2	100	99.76	VFDB	NP_647,403	biofilm and capsule formation
*icaR*	contig_2	100	99.47	VFDB	NP_647,402	biofilm and capsule formation
*Spa*	contig_2	80.32	98.15	VFDB	NP_644,899	immune evasion and stealth
*adsA*	contig_2	100	96.85	VFDB	NP_644,838	immune evasion and stealth
*Sbi*	contig_2	97.11	93.92	VFDB	NP_647,158	immune evasion and stealth
*tsst-1*	contig_2	100	100	VFDB	NP_375,120	toxins and cytotoxins
*lukS-PV*	contig_2	100	99.79	VFDB	NP_646,196	toxins and cytotoxins
*lukF-PV*	contig_2	100	99.9	VFDB	NP_646,195	toxins and cytotoxins
*Sell*	contig_2	100	99.72	VFDB	NP_645,577	toxins and cytotoxins
*Sec*	contig_2	100	97.75	VFDB	NP_645,576	toxins and cytotoxins
*hlgB*	contig_2	100	93.56	VFDB	NP_647,161	toxins and cytotoxins
*hlgC*	contig_2	100	92.93	VFDB	NP_647,160	toxins and cytotoxins
*hlgA*	contig_2	100	99.14	VFDB	NP_647,159	toxins and cytotoxins
*hly/hla*	contig_2	100	95.31	VFDB	NP_645,861	toxins and cytotoxins
*isdF*	contig_2	100	98.97	VFDB	NP_645,833	Iron acquisition
*isdE*	contig_2	100	99.43	VFDB	YP_001332078	Iron acquisition
*isdD*	contig_2	99.91	98.89	VFDB	NP_645,831	Iron acquisition
*isdC*	contig_2	100	99.71	VFDB	YP_001332076	Iron acquisition
*isdA*	contig_2	100	98.77	VFDB	YP_001332075	Iron acquisition
*isdB*	contig_2	99.85	98.71	VFDB	YP_001332074	Iron acquisition
*sdrE*	contig_2	99.47	96.98	VFDB	NP_645,335	adhesion and Surface Proteins
*sdrD*	contig_2	99.75	93.22	VFDB	NP_645,334	adhesion and Surface Proteins
*sdrC*	contig_2	99.51	93.25	VFDB	NP_645,333	adhesion and Surface Proteins
*clfA*	contig_2	98.63	89.66	VFDB	NP_645,581	adhesion and Surface Proteins
*fnbA*	contig_2	99.97	98.79	VFDB	NP_647,238	adhesion and Surface Proteins
*Coa*	contig_2	96.53	81.78	VFDB	NP_645,021	Coagulation

## 4 Experimental Design, Materials, and Methods

### 4.1 Sample collection, isolation, and antimicrobial susceptibility testing

A nasal swab was taken from a milkman handling cows with SCM in the Kiruhura district of Uganda. The nasal sample was cultured on blood agar, and probable isolates were sub-cultured on Mannitol salt agar (Himedia, India) plates, which were incubated aerobically at 37 °C for 24 h. Yellow colonies, indicating *Staphylococcus aureus*, were streaked onto new differential media MSA plates for additional purification. Pure *S. aureus* isolates were further validated by performing positive catalase, coagulase, and Staphaurex latex agglutination tests (Thermo Fisher Scientific) according to the manufacturer's recommendations. [5]. Using the Kirby-Bauer disk diffusion method on Mueller-Hinton agar (MHA) (adjusted to the 0.5 McFarland standard (1.5 × 108 cfu/ml), the *S. aureus* isolate on demonstrated resistance to tetracycline (30 μg; 12 mm), erythromycin (15 μg; 10 mm), penicillin (10 units; 6 mm), and cefoxitin (30 μg, 18 mm) from (Himedia, India), according to 2024 CLSI standards (CLSI M100-ED34th^)^ [6]. Based on its 18 mm zone of inhibition for cefoxitin, the isolate was classified as Methicillin-Resistant *Staphylococcus aureus* (21≤ resistant and ≥22 susceptible).

### 4.2 DNA extraction and whole-genome sequencing

The MRSA isolate was cultured in Luria-Bertani (LB) broth, incubated in a shaker at 200 rpm for 18–24 h at 37  °C, and DNA was extracted using NEB Monarch® Spin gDNA Extraction Kit (New England Biolabs (NEB). DNA concentration and purity were also measured using the Qubit 3.0 Fluorometer with Quant-i­T dsDNA Assay Kit, High Sensitivity (Invitrogen), and Nano Drop ND 1000 spectrophotometer. The MinION MK1B (MIN-101B) (Oxford NANOPORE technology) platform was used for sequencing, producing 1D reads. DNA Library was prepared using the Rapid barcoding kit 96V14(SQK-RBK114.96) (Oxford NANOPORE technologies), and loaded onto an R10 flow cell (FLO-MIN114, Oxford Nanopore Technologies).

### 4.3 Genome assembly and annotation

Raw reads were quality checked with NanoPlot (v1.46.0) before with 18,885.0 total reads, 108,748,143.0 total bases, 11,120.0 read length N50, and 14,217 (75.3%) 96.6Mb >Q10 and after trimming 16,677.0 total reads, 108,188,141.0 total bases, 11,254.0 read length N50, and 14,035 (84.2%) 96.5Mb>Q10 [7]. Adapter sequences and low-quality bases were trimmed using NanoFilt (v2.8.0) [7]. All high-quality trimmed reads were used for de novo assembly with Flye (v2.9.5+galaxy0) [8], and polished with Medaka [9]. All assembled reads passed quality assessment using QUAST (v5.3.0) [10], and assembly completeness was determined using BUSCO (v5.8.3) [11], as shared in Table 1. The assembled genome was annotated using the NCBI Prokaryotic Genome Annotation Pipeline (PGAP v6.10) [12].

### 4.4 AMR gene detection and virulence factor analysis

AMR genes were derived using CARD [13], and ResFinder (v4.6.0)[14]. Likewise, the Virulence Factor Database (VFDB) was used [15] in order to find possible virulence genes.

## Limitations

The dataset is based on a single human nasal isolate, does not include paired isolates from cows, milk, equipment, or the environment.

## Ethics Statement

Both the Uganda National Council for Science and Technology (HS5098ES) and Kampala International University Research Ethics Committee (REC-KIU-2024–436) gave ethical approval. The milkman was given written informed consent before sample collection began.

## CRediT Author Statement

**Reagan Muhwezi**: Conceptualization, Methodology, Supervision, Investigation, Formal Analysis, Writing Original Draft; **Emmanuel Eilu**: Supervision, Conceptualization, Methodology, Review & Editing; **Vidya Sankarapandian:** Software, Data Curation, Visualization; **GR. Neel:** Supervision, Resources, Validation, Software, Formal Analysis, Review & Editing; **Lutaya Festus:** Software, Resources, Validation, Formal Analysis, Review & Editing; **Ambrose Shabohurira:** Resources, Software, Validation, Formal Analysis; **Musoba Abubakar**: Resources, Validation, Formal Analysis, Review & Editing; **Danladi Makeri**: Software, Data Collection, Review & Editing; **Theophilus Pius**: Software, Data Collection, Review & Editing; **Sulman Muhanguzi**: Visualization, Data Curation, Software, Review & Editing; **Naheem Adekilekun Tijani:** Resources, Validation, Software, Project Administration. All authors read and approved the final version of the manuscript.
